# The bioeconomy in Spain as a new economic paradigm: the role of key sectors with different approaches

**DOI:** 10.1007/s10668-022-02830-5

**Published:** 2023-01-05

**Authors:** Valeria Ferreira, Laia Pié, Alfredo Mainar-Causapé, Antonio Terceño

**Affiliations:** 1 Markets and Financial Analysis Research Group, Department of Business Management, Faculty of Business and Economics, Universitat Rovira I Virgili, Av. Universitat n1, 43204 Reus, Spain; 2 Departament of Applied Economics III, Universidad de Sevilla, Avda. Ramón y Cajal 1, 41018 Seville, Spain

**Keywords:** Bioeconomy, Structural analysis, Hypothetical extraction method, Rasmussen, Policy, Implications, Spain

## Abstract

The bioeconomy includes the sectors that use renewable biological resources to produce value added products, such as food, feed, energy, and bio-based products. Its importance has been demonstrated by its inclusion as a priority in specific and related policies such as the bioeconomy strategy, the Sustainable Development Goals, the European Green Deal, and the Next Generation recovery plan. Spain has not lagged behind and considers the bioeconomy as a priority in its policy strategies to achieve a more sustainable economy. Despite its importance, the analysis of the potential of the bioeconomy sectors in Spain is limited. To carry out policy-relevant impact assessment in support of bioeconomy development, specific databases describing bio-based products are required. Hence, this work based on the Bio Social Accounting Matrix (BioSAM) for Spain for the year 2010 with a high disaggregation of bio products to perform a structural analysis based on two different and complementary methods: the traditional and the Hypothetical Extraction approach. The structural analysis results reveal promising products as key wealth generators and growth promoters and allow to identify the most suitable to be stimulated with policies to promote the development of the Spanish bioeconomy. Therefore, this paper provides some proposed avenues that should be considered by policymakers.

## Introduction

The bioeconomy comprises all economic activities based on the use of renewable biological resources to produce food, feed, bioenergy, and bio-based products (European Commission, 2012). This new economic paradigm emerges as the need to focus on a more sustainable economy supporting the achievement of many Sustainable Development Goals (SDGs) (Heimann, 2019). Thus, it has become a priority for the European Union (EU) promoted through its own strategy and related policies such as the European Green Deal or the Next Generation Funds. Specifically, the European bioeconomy strategy was published in 2012 and updated in 2018 (European Commission, 2018). In this sense, there has been a significant interest in the bioeconomy demonstrated by the promotion of related strategies in many countries such as Spain, Finland, Germany, Sweden or Netherlands (Lainez et al., 2018; Priefer et al., 2017), as well as an increasing scientific literature (Keswani et al., 2021). In summary, the bioeconomy offers the possibility of finding new ways of producing the same products based on more efficient use of resources, reducing dependence on non-renewable resources and avoiding resource depletion (European Commission, 2012). All of this while promoting the creation of new products, and having an impact on the competitiveness of enterprises and the generation of new jobs (Lainez et al., 2018).

Despite its importance, little work has been done to analyse the potential of the bioeconomy sectors due to the lack of suitable databases and methodologies. Although different studies have measured the size of the bioeconomy by calculating the biobased shares of the sectors involved (Efken et al., 2016; Ronzon & M’Barek, 2018; Ronzon et al., 2022; Vandermeulen et al., 2011), or using econometric models (Lochhead et al., 2016), the influence of the analysis with multisectoral models, for the analysis of economic, social and/or environmental variables, is clear, for example for Netherlands (Heijman, 2016), Poland (Loizou et al., 2019), Finland (Lehtonen & Okkonen, 2013), Brazil (Maia & Bozelli, 2022), the EU and member states (Ferreira et al., 2020, 2021; Mainar-Causapé, 2019; Philippidis & Sanjuán, 2018). To this end, the Input–Output tables, and the Social Accounting Matrices (SAM) stand out as databases.

With the aim of analysing the potential of bioeconomy products in Spain and promote its development, this article presents on the one hand, a multisectoral structural analysis based on two different and complementary methods: the classical methodology and the Hypothetical Extraction Method (HEM); and, on the other, some policy implications derived from this analysis.

Structural analysis allows us to analyse the linkages between the sectors in an economy and to understand the impacts on wealth generation arising from demand-driven economic shocks. Linkages are defined as the relationships that a sector has with the rest of the economy through the purchase of inputs and the sale of its outputs, showing the sectors that are highly connected and identifying key sectors (Cai & Leung, 2004; Miller & Lahr, 2001). The key sectors are important to promote growth in the economy, as they have the highest potential to stimulate other sectors due to their diffusion capacity in response to final demand and cost variations (Cardenete et al., 2010).

The different methodologies to identify the most important sectors of an economy can be broadly classified into two groups: the classical and the hypothetical extraction approach. In a nutshell, the classical method measures how key the sectors of an economy are, considering the impact they have on other sectors due to demand or supply variations. The HEM determines the significance of a sector by considering the output loss when extracting the sector from the economy (Dietzenbacher et al., 1993). These different methodologies are considered as complementary for a better analysis of the structure of an economy and useful tools for the analysis of ex-ante policy evaluations, (i.e. ‘what if’ questions) (Iráizoz, 2006; Leung & Pooley, 2002; Mainar-Causapé et al., 2020).

The objective of the article is to analyse the bioeconomy structure in Spain using different methodologies and to identify those promising sectors as key wealth generators and growth promoters that will be suitable to stimulate with policies to promote the development of the Spanish bioeconomy. To this end, the symmetric Bioeconomic SAM Matrix for Spain 2010 constructed by the authors is used (Ferreira et al., 2021; Mainar-Causapé et al., 2020), and both the hypothetical extraction method and traditional methodologies are applied.

The empirical application provides an update of the structural analysis of the bioeconomy in Spain and its improvement by using the new bioeconomy database, and different methodologies that have not previously been applied to the analysis of the Spanish bioeconomy. In this way, the BioSAM provides a comprehensive economy-wide frameworks that explicitly represent the linkages between the bioeconomy and the broader macroeconomy, and the combination of methodologies applied enables a more in-depth analysis of these linkages and therefore, a better interpretation of those most suitable sectors for promoting the bioeconomy (Iráizoz, 2006). As the key sector analysis offers the possibility of assess the relative (short-term) wealth-generating impact of a given demand-driven economic shock, it has attracted considerable interest in policy circles to better understand structural economic change and even for policy design (Mainar-Causapé et al., 2020). Consequently, the results obtained in this article could be very useful when designing future strategies that promote the growth of the bioeconomy in Spain since it provides knowledge of which accounts are most suitable for the effective and efficient assignation of government resources. A clear example is related to the allocation of *Next Generation* funds provided by the European Commission, with the aim of recovering the economy because of the crisis generated by Covid-19, which should focus on promoting a sustainable economy.

This article first describes the methodologies for structural analysis and the database used. Then, on section three, the results obtained with each methodology allow for an identification of the most suitable bioeconomy products to be promoted due to their ability to generate wealth. In the discussion section, the results are interpreted considering the contribution of each of the structural analysis methodologies applied. Based on the results, section five includes policy implications and recommendations of the most appropriate products that could be influenced for the development of the Bioeconomy in Spain. Finally, the conclusions of the article are presented in section six.

## Data and methodologies

### Database: bioeconomic social accounting matrix

To focus on the study of the bioeconomy, this article works with a SAM that contains accounts related to bio-based products separated from those that are not. A SAM is a comprehensive and economy-wide database that compiles economic and social information on every transaction made between agents in an economy over a period of time, which is generally one year. The origins of SAM are found in the pioneering works of Stone (1962) and Pyatt and Round (1979), among others.

**Fig. 1:**
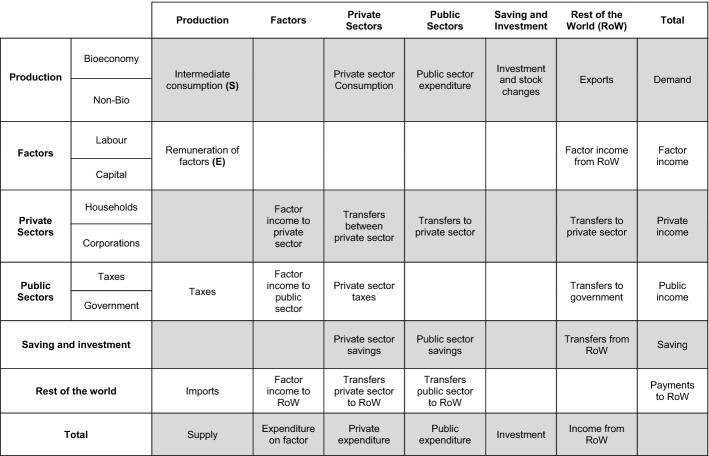
Database basic structure: Bioeconomy SAM symmetric product-by-product. *Source*: based on Mainar et al. (2018) and Ferreira et al. (2021)

Hence, the database used in this article is a symmetric Bioeconomic SAM for Spain 2010 constructed by the authors (detailed in Fig. 1) based on the BioSAM produced for the EU members states (Ferreira et al., 2021; Mainar-Causapé et al., 2020). The bioeconomy SAM obtained has a partial aggregation of 36 products, of which 32 are part of the bioeconomy classified in 4 groups: agriculture, food, biomass, bioindustry, and bioenergy. Coding details of the products presented in the results are clarified in Appendix Table 6, which includes also the detail of all the accounts in the SAM.

### Bioeconomy structural analysis

The idea behind structural analysis is to understand the structure of an economy by analysing the linkages between its sectors to identify those that are highly interconnected and to determine the importance of each sector. Those sectors that prove to have the greatest capacity to stimulate others will be key to promoting the growth of the economy. For this reason, carrying out the structural analysis of an economy is considered an important tool for implementing and evaluating economic policies (Cai & Leung, 2004).

According to the literature, the different methodologies have their advantages and limitations, but to date no single method is considered to be the most appropriate (Leung & Pooley, 2002). While the traditional methodology stands out for its simplicity and reliability, it has the disadvantage of analysing linkages without considering the size of each sector, whereas the HEM allows the size of the sector to be taken into account and has been the most widely used in recent years. Therefore, the application of different methodologies makes it possible to complement the results obtained and, therefore, to analyse the country's structural data in more detail.

The structural analysis of the bioeconomy has previously focused on the use of traditional methods (Mainar-Causapé, 2019; Philippidis & Sanjuán, 2018; Philippidis et al., 2014; Sancho & Cardenete, 2014) and the HEM has been used only for the analysis of the European bioeconomy (Mainar-Causapé et al., 2020). Outside the bioeconomy field, the literature shows many examples where classical and HEM methodologies have been applied in combination, for example to analyse China (Andreosso-O’Callaghan & Yue, 2004), Europe (Mainar-Causapé et al., 2020; Soza-Amigo & Ramos Carvajal, 2005), Spain and its regions (Campoy-Muñoz et al., 2015; Cansino et al., 2013; Cardenete, 2011; Cardenete et al., 2009; Mainar & Flores, 2013), South East Asia (Ali et al., 2019), and Mexico (Boundi, 2017). The following parts of this section will explain the database and the methodologies applied for the structural analysis of the bioeconomy.

#### Traditional methods

The traditional methods analyse the impact that each sector have on the other sectors considering the linkages between them and the direct, indirect, and induced multiplier effects they cause. The traditional methods include the first contributions on structural analysis, published by Rasmussen (1956), and Hirschman (1958) and with some later modifications by other authors such as Augustinovic (1970), Jones (1976) and Beyer (1976).

##### Methodology of Rasmussen (1956) and Hirschman (1958)

The starting point is Leontief's equilibrium equation applied to the case of a SAM as follows ${Y}_{n}={(I-{A}_{nn})}^{-1}{X}_{n}={M}_{nn}{X}_{n}$, where the matrix ${A}_{nn}$ is the coefficient matrix and ${M}_{nn}$ is the multipliers accounting matrix (with elements ${m}_{ij}$). Then, ${Y}_{n}$ represents the income of the endogenous accounts, and ${X}_{n}$ is the final demand of the endogenous accounts represented by government expenditure, investment, and exports.

Rasmussen (1956) proposes obtaining two indexes based on the multipliers obtained in the model. The index of power of dispersion ${{BL}^{R}}_{.j}$ (also called backward linkages, Eq. (1) it represents how a unitary increment in the demand of a sector $j$ is dispersed to the rest of the sectors, measuring the capacity to expand a unitary and exogenous injections of income towards the other endogenous accounts (Andreosso-O’Callaghan & Yue, 2004; Pulido & Fontela, 1993). The sensitivity of dispersion index ${{FL}^{R}}_{i.}$ (also called forward linkage, Eq. (2), shows how the output of a sector $i$ is affected by the increment in the final demand of all the sectors.

**Table:** 

	Total linkages (multiplier effects)		Variation indices
Backward linkage	${U}_{.j }= \sum_{i=1}^{n}{m}_{ij}$ $\forall j=\mathrm{1,2},\dots n$	${{BL}^{R}}_{.j}= \frac{{U}_{.j }}{\frac{1}{n}\sum_{j=1}^{n}{U}_{.j}}$ (1)	${V.}_{j}=\frac{\sqrt{\frac{1}{n-1}\sum_{i=1}^{n}{\left({m}_{ij}-\frac{1}{n}\sum_{i=1}^{n}{m}_{ij}\right)}^{2}}}{\frac{1}{n}\sum_{i=1}^{n}{m}_{ij}}$ $(\forall j=\mathrm{1,2},\dots n)$ (3)
Forward linkage	${U}_{i. }= \sum_{j=1}^{n}{m}_{ij}$ $\forall i=\mathrm{1,2},\dots n$	${{FL}^{R}}_{i.}= = \frac{{U}_{i.}}{\frac{1}{n}\sum_{i=1}^{n}{U}_{i.}}$ (2)	${V}_{i}.=\frac{\sqrt{\frac{1}{n-1}\sum_{j=1}^{n}{\left({m}_{ij}-\frac{1}{n}\sum_{j=1}^{n}{m}_{ij}\right)}^{2}}}{\frac{1}{n}\sum_{j=1}^{n}{m}_{ij}}$ $\left(\forall i=\mathrm{1,2},\dots n\right)$ (4)

Table 1 shows a classification of the sectors according to their BL and FL (Hirschman, 1958) with the aim of determining what the ideal key sectors are for applying economic policies (Andreosso-O’Callaghan & Yue, 2004).

**Table 1:** Classification of sectors according to their backward and forward linkages *Source*: Own elaboration based on Rasmussen (1956)

	BL < Average BL	BL > Average BL
FL < Average FL	Independent sectors	Driving sectors
FL > Average FL	Base sectors	KEY sectors

A key sector requires more inputs than the others in relative terms and provides the rest of the productive sectors with large quantities of intermediate inputs. Therefore, a boost to a key sector stemming from applying an economic policy propagates more extensively to the rest of the economy. A base sector represents a low demand for intermediate inputs, while its outputs that are in great demand by other sectors. This means that the destination of its production serves as inputs for other sectors and, therefore, its variations have important effects on the rest of the sectors. The driving sectors have a huge demand for intermediate inputs from other sectors. In response to exogenous shocks that cause demand for more production they have a huge capacity to drive other sectors, instigating further activities and promoting economic growth. The independent sectors have a lower-than-average incidence in the global economy because their development does not greatly affect the rest of the sectors.

This methodology does not provide information on the concentration of the productive sectors, which may be affected by extreme values. To address the limitation, Rasmussen (1956) proposes the calculation of the dispersion of effects to know whether the effects are spread throughout the economy or whether they are concentrated in certain sectors (Cansino et al., 2013).

Therefore, for a better analysis of the economy structure, ${V.}_{j}$ (Eq. 3) can complement ${{BL}^{R}}_{.j}$, with high value interpreted as an industry whose impact is concentrated in a few sectors and a low value is interpreted as an industry that has a similar influence on all sectors (Hazari, 1970). Thus, ${V}_{i}.$ (Eq. 4) can complement ${{FL}^{R}}_{i.}$ with a low value indicating that the rest of the industries influence $i$ in a similar way, and a high value, indicating that few sectors influence the sector analysed (Soza-Amigo, 2007).

##### Variation in the calculation of FL

One of the major discussions in the literature is the calculation of the FL. Authors like Augustinovic (1970), Jones (1976), Beyer (1976), and Dietzenbacher (1997) consider the Ghosh model to be the most suitable for its calculation. This model is based on the use of the distribution coefficient matrix (${B}_{nn}$) and takes into account value added as an exogenous variable, $Y_{n}^{\prime } = V_{n}^{\prime } \left( {I - B_{nn} } \right) ^{ - 1} = V_{n}^{\prime } G_{nn}$.^1^

With this variation, the effect of changes in the value added of a sector that affect changes in its production can be evaluated (Cardenete et al., 2010; Pulido & Fontela, 1993). Therefore, we can consider that if the FL is higher than average it represents an account with the capacity of cost dispersion because changing the values of its added value has an above average effect on the rest of the sectors (Cardenete et al., 2010). The sectors are classified under the same conditions as for Table 1, with the new FL value.

##### Hypothetical extraction method

The HEM determines the importance of a sector, considering the differences in output upon hypothetically eliminating it from the economy (Dietzenbacher et al., 1993). To this effect, a sector $n$ is extracted from the economy and consequently neither consumes inputs nor sells outputs to the other sectors, impacting on the initial matrix and causing a new output to be obtained (Dietzenbacher et al., 1993). By comparing the level of output for each of the rest of the sectors before and after the hypothetical extraction, it can be seen whether the consequences of the extraction are significant or not for the economy.

Various authors have proposed different ways to “extract” the sector. The review of extraction methodologies published by Miller and Lahr (2001) concludes that the arguments about the virtues and limitations of the methods of extraction can be useless if the aim is to know the importance of a sector (Miller & Lahr, 2001). Consequently, we have chosen to use the application published by Dietzenbacher et al. (1993) recognised as “more evolved and synthesised” (Cardenete & López, 2015) or as the most “paradigmatic” version (Sancho & Cardenete, 2014).^2^

##### Backward linkage

Analysis of the BL serves to indicate the impact of extracting a sector that no longer consumes inputs for its production on the other sectors in terms of reduction in output. Considering the initial equation of Leontief’s demand model:

5 $${Y}_{n}={(I-{A}_{nn})}^{-1}{X}_{n}={L}_{nn}{X}_{n}$$

on hypothetically extracting the sector $j$ the rest of the sectors are represented among the group $r$. Considering the matrix ${A}_{nn}$, partitioned between the sector $j$ and the rest $r$, ${Y}^{j}$ and ${Y}^{r}$ represent the total output of each group, respectively, and ${X}^{j}$ and ${X}^{r}$ represent their respective final demands. On extracting sector $j$, a new matrix of technical coefficients, ${\overline{A} }_{nn}$, and a new output after the extraction represented by $\overline{{Y }^{j}}$ and $\overline{{Y }^{r}}$ are obtained.

6 $${\overline{Y }=(I-{\overline{A} }_{nn})}^{-1}X$$

7 $$\left[\begin{array}{c}\overline{{Y }^{j}}\\ \overline{{Y }^{r}}\end{array}\right]=\left[\begin{array}{cc}{{A}_{n}}^{jj}& 0\\ 0& {{A}_{n}}^{rr}\end{array}\right]\left[\begin{array}{c} \overline{{Y }^{j}}\\ \overline{{Y }^{r}}\end{array}\right]+\left[\begin{array}{c}{X}^{j}\\ {X}^{r}\end{array}\right]$$

8 $$\left[\begin{array}{c}\overline{{Y }^{j}}\\ \overline{{Y }^{r}}\end{array}\right]={\left[\left[\begin{array}{cc}I& 0\\ 0& I\end{array}\right]-\left[\begin{array}{cc}{{A}_{n}}^{jj}& 0\\ 0& {{A}_{n}}^{rr}\end{array}\right]\right]}^{-1}\left[\begin{array}{c}{X}^{j}\\ {X}^{r}\end{array}\right]$$

Total reduction in output on hypothetically extracting sector $j$ is proposed as:

9 $$Y-\overline{Y }=\left(\begin{array}{c} {Y}^{j}-{\overline{Y} }^{j}\\ {Y}^{r}-{\overline{Y} }^{r}\end{array}\right)=\left\{\left[\begin{array}{cc}{{L}_{n}}^{jj}& {{L}_{n}}^{jr}\\ {{L}_{n}}^{rj}& {{L}_{n}}^{rr}\end{array}\right]- \left[\begin{array}{cc}{(I-{{A}_{n}}^{jj})}^{-1} & 0\\ 0& {(I-{{A}_{n}}^{rr})}^{-1} \end{array}\right]\right\}\left[\begin{array}{c}{X}^{j}\\ {X}^{r}\end{array}\right]$$

where $Y$ is the total output prior to extraction and $\overline{Y }$ the value obtained after extracting the sector $j$. Carried out $n$ times, each result constructs a new matrix that collects the backward effects of the extracted sector on the rest of the economy and of the rest of the economy on the extracted sector (Cardenete & Sancho, 2006). The elements that do not belong to the diagonal $i\ne j$ will represent the actual *BL* (Cardenete & López, 2015; Cardenete et al., 2009), which reflects the output lost on the other sector on extracting the sector analysed, given by the backward dependency of the extracted sector on the other sectors (Cardenete et al., 2010; Sancho & Cardenete, 2014).

##### Forward linkage

FL is understood as the impact on output reduction, which in terms of “opportunity cost” the extraction of a sector will have since it will not be able to supply the inputs to satisfy demand from the other sectors (Cansino et al., 2013). This index allows us to know the effect that extracting a sector $j$ has on the rest in terms of sales of their output (Cardenete et al., 2010).

Considering the initial equation of the Ghosh model:

10 $$Y_{n}^{\prime } = V_{n}^{\prime } \left( {I - B_{nn} } \right) ^{ - 1} = V_{n}^{\prime } G_{nn} ,$$

where ${B}_{nn}$ is the distribution matrix, ${V}_{n}$ represents the primary outputs and ${G}_{nn}$ is Ghosh’s inverse matrix. Then, in the partitioned matrix ${B}_{nn}$ the sector to be extracted is separated as $j$ and the rest of the economy is represented by $r$.

On extracting the sector $j$, a new matrix of coefficients of distribution ${\overline{B} }_{nn}$ is obtained, and from this equation a new output is obtained after the extraction represented by $\overline{{Y }^{j}}$ and $\overline{{Y }^{r}}$. Total reduction in output on hypothetically extracting sector $j$ is proposed as $\left( {Y - \overline{Y } } \right)^{\prime }$. Again, sectors are extracted one by one, obtaining new production values for each case and detailing the results of each output in a new matrix. Each element $(i,j)$ will represent the case where the sector was extracted $(j)$, and will represent the forward linkage between the sector $j$ and the sector $i$. The difference between the case with the extracted sector and the original situation reflects the economic losses of the rest of the sectors of the economy without the offer of the extracted sector. The sum of the values outside the diagonal of the matrix obtained are the FL**,** and they quantify the forward relation of the extracted sector $j$ with the global economy.

11 $$\overline{Y}^{\prime} = V^{\prime}\left( {I - \overline{B}_{nn} } \right)^{ - 1}$$

12 $$\left( {\overline{{Y^{j} }} \overline{{Y^{r} }} } \right)^{\prime } = \left[ {V^{j} V^{r} } \right]^{\prime } \left[ {\left[ {\begin{array}{*{20}c} I & 0 \\ 0 & I \\ \end{array} } \right] - \left[ {\begin{array}{*{20}c} {B_{n}^{jj} } & 0 \\ 0 & {B_{n}^{rr} } \\ \end{array} } \right]} \right]^{ - 1}$$

The total output reduction on hypothetically extracting the sector $j$ is proposed as:

13 $$\left( {Y - \overline{Y } } \right)^{\prime } = \left[ {\left( {Y_{j} - \overline{Y}_{j} } \right)^{\prime } { },\left( {Y_{r} - \overline{Y}_{r} } \right)^{\prime } } \right] = \left[ {V^{j} V^{r} } \right]^{\prime } \left\{ {\left[ {\begin{array}{*{20}c} {G_{n}^{jj} } & {G_{n}^{jr} } \\ {G_{n}^{rj} } & {G_{n}^{rr} } \\ \end{array} } \right] - \left[ {\begin{array}{*{20}c} {\left( {I - B_{n}^{jj} } \right)^{ - 1} } & 0 \\ 0 & {\left( {I - B_{n}^{rr} } \right)^{ - 1} } \\ \end{array} } \right]} \right\}$$

## Results

The methods should be used as tools that provides information to describe the structure and linkages between sectors within the Spanish bioeconomy (Cai & Leung, 2004). It provides information on which are the most important sectors to promote the bioeconomy in Spain. Therefore, the approach is useful for descriptive purposes, and these results can be considered preliminary for ex-ante policy decisions (Dietzenbacher & van der Linden, 1997).

### Application of traditional methodology

#### Structural analysis according to Rasmussen/Hirschman

**Fig. 2:**
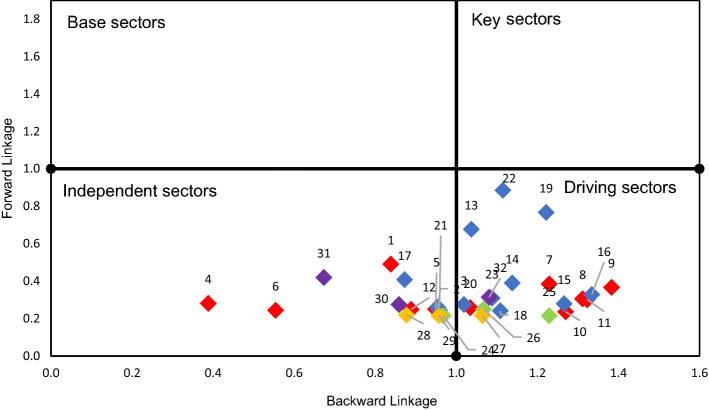
Structural overview of the Bioeconomy sectors, Spain (Rasmussen/Hirschman). Note: Colours by aggregated sector: Agriculture, Food industry, Biomass,  Bioenergy, Bioindustry

The products codes and sectoral classification can be found in Table 6 in Appendix. Table 2 shows the values obtained for each product and their structural classification, which is shown visually in Fig. 2. Their analysis indicates that only services, classified outside the bioeconomy, can be considered as a key product. However, several of the products belonging to the bioeconomy are "driving", able to generate wealth in the rest of the economy because of the linkages they have with other products through the consumption of their inputs. Within the “driving” group, 20 accounts are identified, 19 of which are included in the bioeconomy. Within the bioeconomy, the products included in the agriculture, food industry and biomass groups stand out. As Table 2 shows, when analysing the influence of the bioeconomy products on other accounts through BL, the products related to the livestock, meat, dairy, and beverages sectors stand out.

**Table 2:**
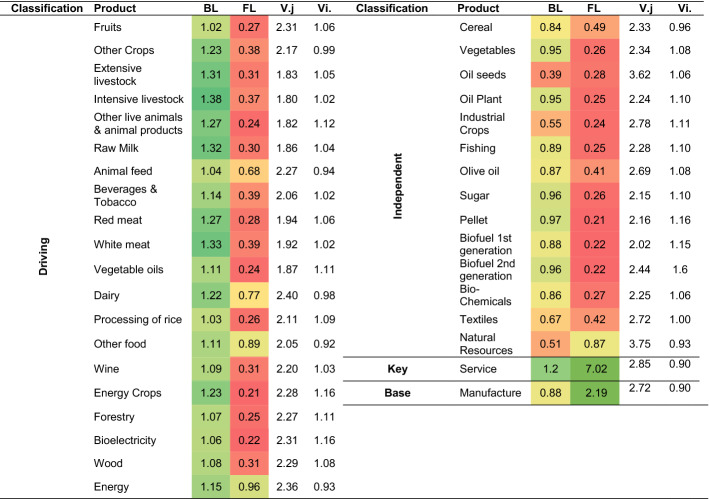
Bioeconomy Product Classification for Spain according to Rasmussen/Hirschman. *Source*: Authors’ own elaboration based on BioSAM Spain 2010 (Ferreira et al., 2021). Note: Values higher than one mean above average. Each value of the BL shows the income expansion effect generated in the endogenous accounts above average due to a unitary exogenous shock of income into the account. Each value of the FL quantifies the increase in income above average in the account as a result of a unit-income exogenous injection in the economy. The gradient colour scale indicates higher values represented by green and lower values by red

For a more in-depth analysis of these results, the calculation of the dispersion effect is considered by ${V.}_{j}$. This coefficient allows us to find that dairy has a high BL, but its origin is concentrated in a few accounts of the economy. On the other hand, products such as extensive and intensive livestock, other live animals and animal products, raw milk, beverages and tobacco, red meat, white meat, and vegetable oil, have a low coefficient of variation, which shows that their backward dispersion effects are distributed throughout the economy.

There are 14 independent accounts, of which 13 belong to the bioeconomy. Hence, these are products that do not use many inputs in their production and most of their supply is concentrated in a few branches or destined for final consumption. Notably, there are 9 products whose BL is higher than 0.85 and therefore close to the group of driving products, which is clearly shown in Fig. 2, for example: vegetables, oil plants, fishing, olive oil, sugar, pellet, first generation biofuel, second generation biofuel, and biochemicals. However, the BL analysis of these products classified in the bioindustry, and bioenergy groups shows that the above-average and below-average values are not significant.

In the base product group, no bioeconomic product stands out, and only the manufacturing product category is greater than one. Considering only the average of the bioeconomy products to avoid the distortion caused by the high values of a few accounts, the FL of bioeconomic products also tend to be lower than the average for most of the products. That means that the supply chain of bioeconomy products is less spread among accounts, concentrating sales in some of them and obtaining low multiplier effects. This can also be seen when considering the values of ${V}_{i}.$, whose high values indicate that the effects are not dispersed across the rest of the economy. However, there are some products previously classified under independent, whose FL values are above of the bioeconomy products average and with low ${V}_{i}.$, demonstrating that some Bioeconomy products have a higher FL, being products that are an essential input for other food processing activities (e.g., cereals, other crops, animal feed, olive oil, dairy, and other foods products).

#### Structural analysis according to Rasmussen and the variation of FL

**Fig. 3:**
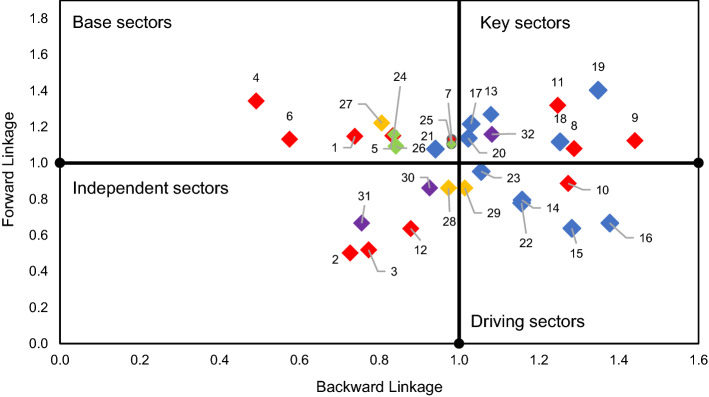
Structural overview of the Bioeconomy sectors, Spain (Rasmussen and the FL variation). Note: Colours by aggregated sector: Agriculture, Food industry, Biomass, Bioenergy, Bioindustry

Table 3 presents the values obtained for each product and their structural classification, which is illustrated in Fig. 3. The analysis of the results of the FL considering Ghosh's inverse matrix indicates that there are 10 key products, of which 9 belong to the bioeconomy, highlighting those included in agriculture and the food industry. There are 8 driver products, 7 of which are part of the bioeconomy and are mainly in the food industry, as well as in agriculture and also within bioenergy as second generation biofuels.

**Table 3:**
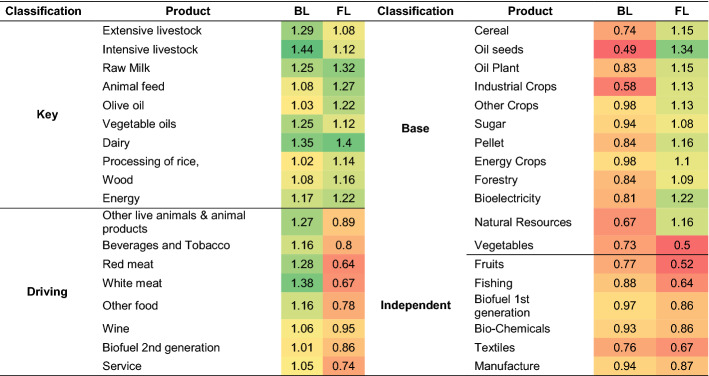
Bioeconomy Product Classification for Spain according to Rasmussen with FL variation *Source*: Author’s’ own elaboration based on BioSAM Spain 2010 (Ferreira et al., 2021). Note: Values higher than one mean above average. The gradient colour scale indicates higher values represented by green and lower values by red

Eleven products are classified as base products. Those that are part of the bioeconomy mainly belong to agriculture and biomass and in bioenergy there is only bioelectricity. Based on the results, 7 products are classified as independent, with vegetables, fruits, fishing, 1st generation biofuels, bio-chemicals, and textiles within the bioeconomy. Regarding the interpretation of the FL, the different results are due to the difference in its calculation and the matrices used. The calculation of the FL according to the variation explained, considers the matrix of coefficients outputs, taking into account the value added as an exogenous account. This index indicates how the outputs of a product are distributed, therefore, those products that are highly demanded by others will have a high FL. However, the FL calculated according to Rasmussen is based on the input coefficient matrix, and it will be high in cases where the output of a product is used by several endogenous accounts. This implies, for example, that a sector whose products are in high demand as intermediate consumption, but concentrated in few branches, will have a low FL according to Rasmussen, but it will be high when calculated with the presented variation (e.g. Oil seeds).

### Hypothetical extraction of bioeconomy products

#### Backward linkage results

The BL results show the output lost in the economy when a product is extracted, due to the intersectoral effects produced by the dependence of the extracted product on the other products. This impact generated by the extraction of the sector is represented in relative values in Table 4, considering the total value of the rest of the products (column 4) and the value extracted (column 5).

**Table 4:** Results of the Hypothetical Extraction of sectors. *Source*: Author’s own elaboration based on BioSAM Spain 2010 (Ferreira et al., 2021)

Group	Product	Amount extracted	BL		FL	
			BL/Total- Amount extracted (%)	BL/Amount extracted (%)	FL/Total- Amount extracted (%)	FL/Amount extracted (%)
Agriculture	Cereal	6161	0.25	95.92	0.41	**153.80**
	Vegetables	8864	0.36	94.70	0.05	12.05
	Fruits	8672	0.40	107.53	0.06	16.17
	Oil seeds	1776	0.02	30.77	0.15	**197.18**
	Oil Plant	1063	0.06	124.36	0.07	**157.50**
	Industrial Crops	425	0.01	54.82	0.03	**154.41**
	Other Crops	5671	0.40	**163.24**	0.37	**153.08**
	Extensive livestock	3402	0.36	**244.50**	0.21	**140.45**
	Intensive livestock	7662	0.85	**258.31**	0.44	**133.36**
	Other live animals & animal products	1372	0.14	**241.25**	0.06	97.96
	Raw Milk	2297	0.23	**231.03**	0.19	**191.61**
	Fishing	3532	0.20	132.07	0.06	40.18
Food	Animal feed	14,782	0.95	**149.21**	0.93	**144.72**
	Beverages & Tobacco	16,187	1.12	**160.29**	0.35	49.84
	Red meat	9275	0.97	**241.74**	0.17	42.35
	White meat	15,765	**1.80**	**263.43**	0.33	48.02
	Olive oil	7010	0.23	76.12	0.22	73.11
	Vegetable oils	2516	0.26	**236.78**	0.16	**149.61**
	Dairy	13,015	0.55	97.31	0.40	71.43
	Processing of rice	784	0.06	**175.11**	0.05	**153.98**
	Sugar	1021	0.07	**152.91**	0.06	**140.74**
	Other food	47,558	**3.44**	**164.69**	1.08	51.72
	Wine	8458	0.66	**181.55**	0.41	112.36
Biomass	Pellet	3	0.0001	125.15	0.0012	**1075.83**
	Energy Crops	5	0.0004	**164.10**	0.0004	**175.66**
	Forestry	1322	0.07	118.00	0.08	**135.06**
Bioenergy	Bioelectricity	126	0.01	116.93	0.01	**171.32**
	Biofuel 1st generation	626	0.06	**217.78**	0.02	91.19
	Biofuel 2nd generation	142	0.01	**173.05**	0.01	89.76
Bioindustry	Bio-Chemicals	11,213	0.72	**148.02**	0.45	92.25
	Textiles	35,346	1.11	72.17	0.42	27.32
	Wood products	8035	0.50	143.38	0.41	117.06
Non-Bio	Natural Resources	86,023	1.21	31.41	**3.42**	89.15
	Energy	80,118	**3.74**	104.89	**2.92**	81.86
	Manufacture	450,694	**17.20**	71.60	**9.59**	39.91
	Service	1,466,186	**50.51**	29.66	**21.69**	12.74
	Total	2.327.107				

For each product, the value that was extracted and the impact it represents on the economy after extraction is presented, considering backward and forward effects. Higher values are Bold

Thus, considering column 4, the extracted products with greater weight in the economy stand out. Consequently, those of the non-bioeconomy and food industry groups predominate. Next are other food products and white meat, both of which belong to the bioeconomy within the food industry group (shaded in column 4 in Table 4). To analyse the products within the bioeconomy, the impact of the extraction of each of its products on the total available in the bioeconomy is considered. Thus, the results indicate that intensive livestock stands out within agriculture, and within the food industry mainly other food and white meat but also animal feed, red meat, diary, wine and beverages, and tobacco. In the bioindustry, textiles and biochemicals stand out, in addition to low value wood products. The least important considering the average of the bioeconomy are first generation biofuels, oilseeds, oil plants, second generation biofuels, industrial crops, bioelectricity, energy crops and pellets, most of which belong to the bioenergy and biomass groups.

Column 5 indicates the percentage decrease in the output of the other sectors in proportion to the output that was extracted (Dietzenbacher & van der Linden, 1997; Dietzenbacher et al., 1993). These relative values enable the size effect to be corrected, identifying whether there are products of low value but whose impact is much higher compared to the amount extracted. In this case, mainly the products with strong linkages with the others will stand out, which is why the results obtained are similar to the analysis of the BL using the traditional methodology. Considering this relative value, non-bioeconomy products do not stand out.

#### Forward linkages results

The results of the FL in relative values are shown in columns 6 and 7 of Table 4. The relative results as percentages of the rest of the economy's output are represented in column 6 (Miller & Lahr, 2001). This means that the highest values are related to the products with the highest output, and the results considering the average of all sectors indicate that only non-bioeconomy products stand out. If the results are analysed considering the average of the bioeconomy products, those within agriculture stand out, including cereals, other crops, and intensive livestock; within the food industry, animal feed, beverages and tobacco, white meat, dairy, other food, and wine; and in the bioindustry, biochemicals, textiles, and wood products.

In column 7, the FL results indicate the percentage by which the output of the other products decreases as a proportion of the extracted output (Dietzenbacher & van der Linden, 1997; Dietzenbacher et al., 1993). In this case, considering the average of all products, the most important within agriculture are cereals, oilseeds, oilseed plants, industrial crops, other crops, extensive livestock, intensive livestock, and raw milk; within the food industry, animal feed, vegetables oil, rice and sugar; within the biomass group, pellets, energy crops and forestry; and within bioenergy, bioelectricity. There is no single product that stands out either within the bioindustry or the non-bioeconomy.

## Discussions

The application of the different methodologies and their results show that there are bioeconomy products in Spain that stand out due to their quantitative importance in the rest of the economy and others that stand out because of their quantitative relevance and/or because they have significant links with the rest of the accounts. Both must be taken into account when taking policy decisions to promote them. This justifies the importance of discussing them considering the combination of the methodologies applied and analysing how the influence of each product is interpreted according to each of them. The summary of the classification of each bioeconomy product considering the three methodologies are detailed in Table 7 in the Appendix.

In the bioeconomy in Spain, the products related to the food industry stand out for their quantitative importance and their links with the other accounts. However, considering the average of those belonging to the bioeconomy,^3^ the results with the HEM show that various products mainly included in the food industry and bioindustry sectors, and one product in the agricultural sector, are important as much for their representative values in the bioeconomy as for their links with the other accounts. Intensive livestock, animal food, white meat, dairy products, beverages (including wine), tobacco, and other food products stand out. In the bioindustry, textiles, biochemicals and other wood products stand out.

Furthermore, of the products mentioned, those related to agriculture and the food industry are very linked to other sectors. This is because they use greater quantities of inputs than the other products for their production and they are also in demand as intermediate inputs for the other products or for households. Similar result was obtained for the analysis of the bioeconomy in Europe (Philippidis & Sanjuán, 2018; Philippidis et al., 2014), the authors of which explain that, for example in the agricultural sector, is due to the intensive demand of inputs required for production (fertilisers, transport-related services, veterinary, machinery, energy, etc.).

Thanks to the different methodologies applied, it is also possible to differentiate the products whose value in the economy is not significant but whose relationships with the other accounts are important and so can have an influence on the sustainable development of the economy. It is important to highlight the importance of the bioeconomy products, mainly due to their backward links with the higher BL values. This means that the bioeconomy contains products that use various inputs for their production and are also influenced by work and capital. Agriculture, the food industry, the supply of biomass, bioelectricity, and wood products mainly stand out. Within agriculture and the food industry, apart from the products previously mentioned due to their quantitative importance, we can add as relevant other live animals and products of animal origin, raw milk, rice, vegetable oils, and other crops due to their links with red meat production.

Similarly, analysis of the forward links of the bioeconomy products shows that various of them within agriculture, the food industry and biomass, bioelectricity, and wood products are internally demanded by the other branches to produce. However, comparison of the FL calculations indicates that the distribution of the outputs of the products of the bioeconomy is centred on just a few branches, limiting the dispersion of the effects. According to the results obtained for the bioeconomy in Europe, these products require less support from others to process and distribute one unit to the end-users (Philippidis & Sanjuán, 2018; Philippidis et al., 2014).

Continuing with the interpretation of the results, within bioenergy, biofuels are considered as independent products. Although their backward links are close to the average, their forward links are low because their intermediate demand is mainly focused on manufacturing and services, and the final demand is mainly focused on exports and households.

We can also discern the products that stood out previously due to their quantitative importance but whose links with the other accounts represent a below average influence, mainly within the bioindustry, textiles, bio-chemicals and wood products. Nonetheless, exclusively analysing their links with the other accounts reveals that only wood products are considered as a product with important relations driving the growth of the economy to an above average extent in terms of sectors, with the influence of work and capital standing out. Both textiles and biochemicals lack higher than the economy average links with the other accounts, considered as independent branches. These products have an important offer in the bioeconomy, which stands out for its imports.

## Policy implications

Due to the current situation of climate change and the health emergency, the bioeconomy has become a necessity and opportunity to drive an economic recovery with a sustainable approach. In this regard, for the adequate use of *Next Generation* funds, Spain has drawn up the *Recovery, Transformation and Resilience Plan*. This plan aims to select strategic sectors with a high capacity to drive economic growth and employment in Spain, while seeking to promote the bioeconomy and the ecological transition. For this reason, it is essential to analyse the situation of the Spanish bioeconomy and to know which key sectors should receive political support funds.

Considering that our objective is to highlight those sectors of the Bioeconomy that have a greater impact on economic growth in general and on the growth of the Bioeconomy in particular, the summary of Table 7 allows us to interpret the tendency of bioeconomic products to behave in a certain way if policy decisions were applied to them. In this sense, the analysis of the linkages shows the importance of the bioeconomy products’ relationships with the other accounts because most of them can be considered as driver, base or key products within the economy. This indicates that an exogenous injection in their demand or primary inputs can rebound on the rest of the economy to a greater degree than on the rest of the sectors.

The methods applied allows us to be mainly affirmed that various bioeconomy products are important both for the total they represent in it and for the relationship they have with the other accounts. Hence, this article concludes that to promote the Spanish bioeconomy, the products with the greatest influence identified in this study as "key sectors" by some methodology must be stimulated. As a summary, Table 5 shows the case of those bioeconomy products classify as key sector by at least one methodology. These are mainly other crops (7), extensive (8), and intensive livestock and products (9), and raw milk (11) -in the agriculture group-, considering the food industry animal feed (13), vegetable oils (18), olive oil (17), sugar (21), rice (20), and dairy (19). Within the biomass group only energy crops (25) and only wood products (32) within the bioindustry group. As a result, given an initial shock to the economy, each of these products has influence to generate a cumulative demand-driven (backward-linkage) and supply-driven (forward-linkage) wealth effect. This means that they are products whose influence on the rest of the accounts is above average and, therefore, they can be considered strategic for the growth of the economy. For example, dairy products use many inputs in their production and their outputs are also highly demanded, generating multiplier effects in the economy.

**Table 5:** Bioeconomy products classify as key sector by at least one methodology

Product	HEM**	Rasmussen/FL variation
Other crops	K	B
Extensive livestock	K	K
Intensive livestock	K	K
Raw milk	K	K
Animal feed	K	K
Olive oil	I	K
Vegetable oils	K	K
Dairy	I	K
Processing of rice. milled or husked	K	K
Sugar	K	B
Energy Crops	K	B
Wood products	I	K

K, key sectors; B, base sectors, I, independent sectors

**Relative value according to the amount extracted

Despite not being considered as key products, the analysis of the products identified as driving or base also allows policy recommendations to be made on them. Considering the biomass group of products, they are mostly considered as base sectors because they are forward oriented as they are products widely used by other sectors. Further promoting its use as a raw material for the biochemical and bioenergy industry is essential to achieve the objectives of the Spanish Bioeconomy Strategy Horizon 2030.

From a policy point of view, the primary sector in Spain, have the potential to promote economic growth in the rest of the economy sectors. Hence, up to this point, policies should focus on promoting Spain bioeconomy more traditional sectors because it can help to promote the growth of the bioeconomy thought the relations with other sectors. Furthermore, the promotion of agriculture will influence the development of rural areas, which will have an impact on SDGs such as food security and job creation, also helping to achieve the ‘*Empty Spain*’^4^ goal. As the policy implications for bio-economic products will have a global effect on rural areas, this should, as far as possible, be adopted in a coordinated manner.

When analysing those sectors of the bioeconomy that can be considered as ‘modern’ (i.e.bioindustry and bioenergy), it can be observed that although some of them do not represent a significant value within the bioeconomy in Spain, their linkages indicate that they can be considered as drivers or base in most cases. This is because they are capable of generating above-average demand-driven effects (bioelectricity) or economic activity very close to the average (biofuels, bio-chemicals, and textiles) in the rest of the economy due to their demand for inputs from other branches and their use of capital and labour.

Therefore, the promotion of these products will mean that they will represent a larger quantity in the bioeconomy and that their backward and forward linkages promote the development of other activities within it, promoting economic growth. In the case of biofuels, the Spanish policy aims to reach 10% of biofuels consumption by 2022. However, considering our analysis it can be said that in addition to the identified exports, their use by households and industries should be encouraged to influence the promotion of the sector. Another point is the case of the bioindustry, both textile and biochemical, which represent a high supply in the bioeconomy due to import dependency. This implies that Spain should encourage the production of such biobased products, generating national economic growth and employment.

Consequently, the transition to a biobased economy requires that public investment R + D + i and political support focus on the most innovative biobased products. As the bioeconomy is based on the creation of an economy that uses biological resources and avoids fossil-based resources, it is essential to invest in innovation and new technologies to improve existing value chains and create new ways of bio-based production. In so doing, production for their products increases, having a knock-on effect on the rest of the economy growth and employment. In addition, increased production must be accompanied by policies that enable market penetration and favour the use of products with greater biobased content. For example, there could be market incentives for commercialization of biobased products, benefits for companies that use renewable energy and bio-based inputs, or policies that set mandatory percentages for the consumption and sale of some bio-based products.

In summary, the bioeconomy in Spain is a recent paradigm, which is intended to be promoted since the publication of its strategy in 2016: ‘Spanish Bioeconomy Strategy: Horizon 2030’. Therefore, it will be important to invest not only in the traditional sectors previously mentioned that stand out as important in the economy, but also in those that are important at strategic levels because they enable the expansion of the products belonging to the bioeconomy, thus aiding its development in Spain. These results will support one of the main lines of the Spanish bioeconomy strategy, which considers public and private research and collaborations and business investment in innovation in the bioeconomy to be essential (Lainez et al., 2018).

## Conclusions

In recent years, the bioeconomy has emerged as a new economic paradigm which offers the possibility of finding new ways of supplying the same products based on the more efficient use of resources, reducing dependence on non-renewable resources and avoiding resource depletion. It is also based on promoting the creation of new products, developing new economic activities, increasing companies’ competitiveness and thereby generating new jobs (European Commission, 2012).

For this reason, it is essential to analyse the situation of the Spanish bioeconomy and to find out about possible actions that could help to promote it. In this way, it is necessary to work with a suitable database that can be used for economic modelling. Therefore, this article presents a structural analysis of the bioeconomy sectors in Spain based on the BioSAM. The significance of this matrix is based mainly on the split of several products within the bioeconomy. Considering the bioeconomy sectors, this article focuses on a new perspective and compliment the structural analysis applying different methodologies with the purpose of examining in-depth the relations within the sectors.

In other words, different methodologies have been applied to the BioSAM to analyse the structure of the bioeconomy in Spain and to understand the potential of each bio commodity to generate influence in the rest of the economy, providing a complete analysis of Spanish Bioeconomy. The main purpose of the methodologies applied is to provide more detailed information about the links between the accounts of the Spanish bioeconomy. The descriptive analysis applied allowed us to identify which products stand out most for their economic importance and/or their link with the other accounts, promoting the development of the rest, and also the products whose economic maturity is not yet relevant. This gives the possibility of finding out possible economic drivers that will have the greatest reach and capacity to promote the bioeconomy. Not only can the key products be identified, but also the products which, although they no longer stand out in the market, can be considered as strategic in the development of the bioeconomy because of their relationships with the rest of the accounts and because they are priority for the bioeconomy itself.

The results indicate that the more traditional bioeconomy products (such as primary sector) have the potential to promote economic growth in the rest of the economy sectors since they stand out for their importance and/or their relations with the other accounts. Special attention should be paid to those other more ‘modern’ products included in this new matrix, particularly in the bioenergy and bioindustry groups. In the first case, neither biofuels nor bioelectricity represent a significant value in the Spanish bioeconomy. Furthermore, biofuels stand out for their importation and not for their linkages with capital and work. In the case of the bioindustry, it can be observed that their quantity within the bioeconomy is mainly representative due to the influence of textiles. However, both textiles and bio-chemicals also stand out for their imports, while they do not represent important linkages with the other accounts in the economy. This means that the bioeconomy products considered as more ‘modern’ have not reached their maximum potential in the Spanish bioeconomy.

The methodology is a useful tool which allows for an analysis of an economy structure and ex-ante policy assessments. Nevertheless, the different structural analysis methodologies applied work with the Leontief demand model and Ghosh offer model, with their respective limitations (Soza-Amigo & Ramos Carvajal, 2005). These limitations are mainly related to the excess capacity in all sectors and unemployed factors of production (no supply constraints) and fixed prices (not taking substitution effects into account), among others (Miller & Blair, 2009). Therefore, it should be noted that this study cannot address the analysis of the transformation of input use in industries and includes data from 2010.

Last and notably, we identified the key or strategic products to promote the Spanish bioeconomy exclusively considering the economic aspect and without considering environmental variable, such a greenhouse gas emissions or land use change (Mougenot & Doussoulin, 2021). As the goal is sustainable development, the bioeconomy should consider not only economic, but also social and environmental impact analysis (Ramcilovic-Suominen & Pülzl, 2018). Therefore, to make comprehensive policy recommendations, future lines of research should focus on the assessment of the bioeconomy, considering also environmental impact. In addition, the impact of the COVID-19 restriction and also the conflict between Ukraine and Russia have important implications on agricultural and food systems and thus on the bioeconomy. Therefore, this could also be an important focus for future research.

## Appendix

See Tables

**Table 6:** Details of the accounts in the BioSAM including products coding. *Source*: Authors’ own elaboration based on the BioSAM (Mainar et al., 2020)

Products		
Aggregated	Code	Account
Primary sector	1	Cereal
	2	Vegetables
	3	Fruits
	4	Oil seeds
	5	Oil plant
	6	Industrial crops
	7	Other crops
	8	Extensive livestock & products
	9	Intensive livestock & products
	10	Other live animals & animal products
	11	Raw Milk
	12	Fishing
Food	13	Animal feed
	14	Beverages & tobacco
	15	Red meat
	16	White meat
	17	Olive oil
	18	Vegetable oils
	19	Dairy
	20	Processing of rice. milled or husked
	21	Sugar
	22	Other food
	23	Wine
Biomass	24	Pellet
	25	Energy crops
	26	Forestry
Bioenergy	27	Bioelectricity
	28	Biofuel 1st generation
	29	Biofuel 2nd generation
Bioindustry	30	Bio-Chemicals
	31	Textiles
	32	Wood products
Non Bioeconomy	33	Natural resources
	34	Energy
	35	Manufacture
	36	Service

Other accounts	
Factors of production	Labour
	Capital
Taxes	Net production taxes
	Net products taxes
	Direct taxes
Private and public agents	Households
	Enterprises/Corporations
	Government
Capital account	Investment-savings
External relations	Rest of the world

6 and

**Table 7:** Structural classification of products according to each methodology. *Source*: Author’s own elaboration based on BioSAM Spain 2010 (Ferreira et al., 2021)

Product	Total economy				Average of bioeconomy products			
	HEM*	HEM**	Rasmussen	Rasmussen /FL variation	HEM*	HEM**	Rasmussen	Rasmussen /FL variation
Cereal	I	B	I	B	B	B	B	B
Vegetables	I	I	I	I	I	I	I	I
Fruits	I	I	D	I	I	I	D	I
Oil seeds	I	B	I	B	I	B	I	B
Oil Plant	I	B	I	B	I	B	I	B
Industrial Crops	I	B	I	B	I	B	I	B
Other Crops	I	K	D	B	B	K	K	B
Extensive livestock	I	K	D	K	I	K	D	K
Intensive livestock	I	K	D	K	K	D	K	K
Other live animals & animal products	I	D	D	D	I	D	D	D
Raw Milk	I	K	D	K	I	K	D	K
Fishing	I	I	I	I	I	I	I	I
Animal feed	I	K	D	K	K	B	K	K
Beverages & Tobacco	I	D	D	D	K	D	K	D
Red meat	I	D	D	D	D	D	D	D
White meat	I	D	D	D	K	D	D	D
Olive oil	I	I	I	K	I	I	B	K
Vegetable oils	I	K	D	K	I	K	D	K
Dairy	I	I	D	K	K	I	K	K
Processing of rice. milled or husked	I	K	D	K	I	K	D	K
Sugar	I	K	I	B	I	B	I	B
Other food	D	D	D	D	K	D	K	D
Wine	I	D	D	D	K	D	D	D
Pellet	I	B	I	B	I	B	I	B
Energy Crops	I	K	D	B	I	K	D	B
Forestry	I	B	D	B	I	I	D	B
Bioelectricity	I	B	D	B	I	B	D	B
Biofuel 1st generation	I	D	I	I	I	D	I	I
Biofuel 2nd generation	I	D	I	D	I	D	I	D
Bio-Chemicals	I	D	I	I	K	I	I	I
Textiles	I	I	I	I	K	I	B	I
Wood products	I	I	D	K	K	I	D	K
Natural Resources	B	I	I	B	K	I	I	B
Energy	K	I	D	K	K	I	D	K
Manufacture	K	I	B	I	K	I	B	I
Service	K	I	K	D	K	I	K	D

K, key sectors; D, driving sectors, B, base sectors, I, independent sectors. * Relative value according to the total economy without the amount extracted. ** Relative value according to the amount extracted

7

## Footnotes

1 For more detail see Dietzenbacher (1997)

2 Cardenete and Sancho (2006) later extended its use to a SAM (Cardenete and Sancho 2006; Sancho and Cardenete 2014), which has been applied in different research (Beltrán et al., 2016; Cansino et al., 2013; Cardenete 2011; Cardenete and López 2015; Cardenete et al., 2009).

3 It is considered appropriate to compare the values of each product of the bioeconomy with the average of its products. In this way, it is possible to analyse just the bioeconomy, leaving aside the non-bio products that distort the analysis due to their quantitative importance.

4 It refers to the territories in Spain affected by the depopulation of rural areas.
